# Quantifying innervation facilitated by deep learning in wound healing

**DOI:** 10.1038/s41598-023-42743-5

**Published:** 2023-10-06

**Authors:** Abijeet Singh Mehta, Sam Teymoori, Cynthia Recendez, Daniel Fregoso, Anthony Gallegos, Hsin-Ya Yang, Elham Aslankoohi, Marco Rolandi, Roslyn Rivkah Isseroff, Min Zhao, Marcella Gomez

**Affiliations:** 1 Department of Dermatology, University of California, Davis, CA 95616 USA; 2 Department of Ophthalmology, University of California, Davis, CA 95616 USA; 3 Department of Applied Mathematics, University of California, Santa Cruz, CA 95064 USA; 4 Department of Electrical and Computer Engineering, University of California, Santa Cruz, CA 95064 USA

**Keywords:** Biological techniques, Molecular biology

## Abstract

The peripheral nerves (PNs) innervate the dermis and epidermis, and are suggested to play an important role in wound healing. Several methods to quantify skin innervation during wound healing have been reported. Those usually require multiple observers, are complex and labor-intensive, and the noise/background associated with the immunohistochemistry (IHC) images could cause quantification errors/user bias. In this study, we employed the state-of-the-art deep neural network, Denoising Convolutional Neural Network (DnCNN), to perform pre-processing and effectively reduce the noise in the IHC images. Additionally, we utilized an automated image analysis tool, assisted by Matlab, to accurately determine the extent of skin innervation during various stages of wound healing. The 8 mm wound is generated using a circular biopsy punch in the wild-type mouse. Skin samples were collected on days 3, 7, 10 and 15, and sections from paraffin-embedded tissues were stained against pan-neuronal marker- protein-gene-product 9.5 (PGP 9.5) antibody. On day 3 and day 7, negligible nerve fibers were present throughout the wound with few only on the lateral boundaries of the wound. On day 10, a slight increase in nerve fiber density appeared, which significantly increased on day 15. Importantly, we found a positive correlation (R^2^ = 0.926) between nerve fiber density and re-epithelization, suggesting an association between re-innervation and re-epithelization. These results established a quantitative time course of re-innervation in wound healing, and the automated image analysis method offers a novel and useful tool to facilitate the quantification of innervation in the skin and other tissues.

## Introduction

Wound regeneration is a complex process that is regulated by orchestrated mechanisms, influenced by chemical, cellular, and molecular factors^1,2^. The healing process begins at the time of injury and eventual maturation could continue for months or even years until the wound completely heals and is structurally and functionally similar to uninjured skin^3^. The four overlapping phases of wound healing are homeostatic, inflammatory, proliferative, and remodeling. The homeostatic phase lasts a few hours producing a fibrin plug followed by an inflammatory phase, which can last between hours and days, during which aggregated platelets and cells release pro-inflammatory mediators^4^. The early inflammatory phase is succeeded by the proliferative phase lasting a few weeks during which macrophages and fibroblast cells invade the wound bed forming granulation tissue and active migration of the wound epithelial cells occurs^5^. The last phase of wound healing is the remodeling phase, which is characterized by proliferative cell apoptosis, adjustment of extracellular matrix (ECM), and replacement of type 3 with type 1 collagen in the dermis. It can last for weeks to years^3^.

During healing phases, a strong interaction between the nervous system and skin involving a variety of neuromodulators, cytokines, hormones, and other effector molecules has been reported^6,7^. The nervous system can be influenced both at the local and central levels by the stimuli at the skin and vice versa. The brain can alter skin function during the pathophysiological state and skin can modulate the nervous system by releasing a variety of neuropeptides^8,9^. The cutaneous nerves therefore positively affect all the stages of wound healing^10^. Numerous neuropeptides e.g. substance P (SP) released from cutaneous nerves have been reported to activate vital mechanisms during the inflammatory phase^11^. Similarly, neuropeptides released by cutaneous nerves influence the proliferation phase. They can promote the proliferation of fibroblasts, keratinocytes, and endothelial cells by stimulating DNA synthesis, can stimulate angiogenesis, support granulation tissue remodeling, and many more^5,12–15^. The effect of innervation on the remodeling stage has also been studied in the past. It has been demonstrated that a significantly higher number of nerve fibers correlate with normotrophic scars in comparison to hypertrophic scars during the remodeling phase^16,17^. Hence, the literature strongly suggests a regulatory role for skin nerves in wound healing and any impairment in skin innervation is one of the leading causes of occurrence of chronic wounds e.g., diabetic neuropathy can lead to foot ulcers and plegias to sacral and trochanteric pressure sores^18,19^.

Previously, numerous studies have been conducted on quantifying skin innervation^20–28^. However, most such studies are not fully automated, have manual counts of IHC-stained structures that are prone to user errors and variation, require multiple observers, and are complex and labor-intensive. One such example is dendrite analysis involving manually tracing neurons using the simple neurite tracer plug-in of ImageJ software^21,22^. While this semi-manual approach has been proven effective, it involves identifying the beginning and end points of dendrites and digitally drawing individual branch segments throughout the entire neuron, making it a labor-intensive and time-consuming process. Another example is semi-automated Sholl analysis for quantifying changes in the growth and differentiation of neurons and glia^23^. The method offers several advantages over conventional manual quantification, including faster analysis time and increased statistical sensitivity. However, the method has some limitations, such as reliance on manual input from the user, which introduces a risk of user error and variation impacting the accuracy and reliability of the results. Additionally, the semi-automated Sholl method is complex and time-consuming to set up initially, which could act as a barrier for researchers who do not have the technical expertise or resources to implement the method effectively. In an effort to quickly, objectively, and reproducibly quantify cutaneous innervation we developed a fully automated Matlab-assisted image analysis tool aided by the deep neural network, DnCNN, for pre-processing (de-noising) of the IHC-images. This network can detect and remove high-frequency image artifacts and increase image resolution Image noise is minimized, resulting in higher quality images that can be more accurately analyzed. The DnCNN is particularly developed for image processing^29^, and has shown effectiveness in a wide range of applications, including medical imaging^30^.

**Figure 1:**
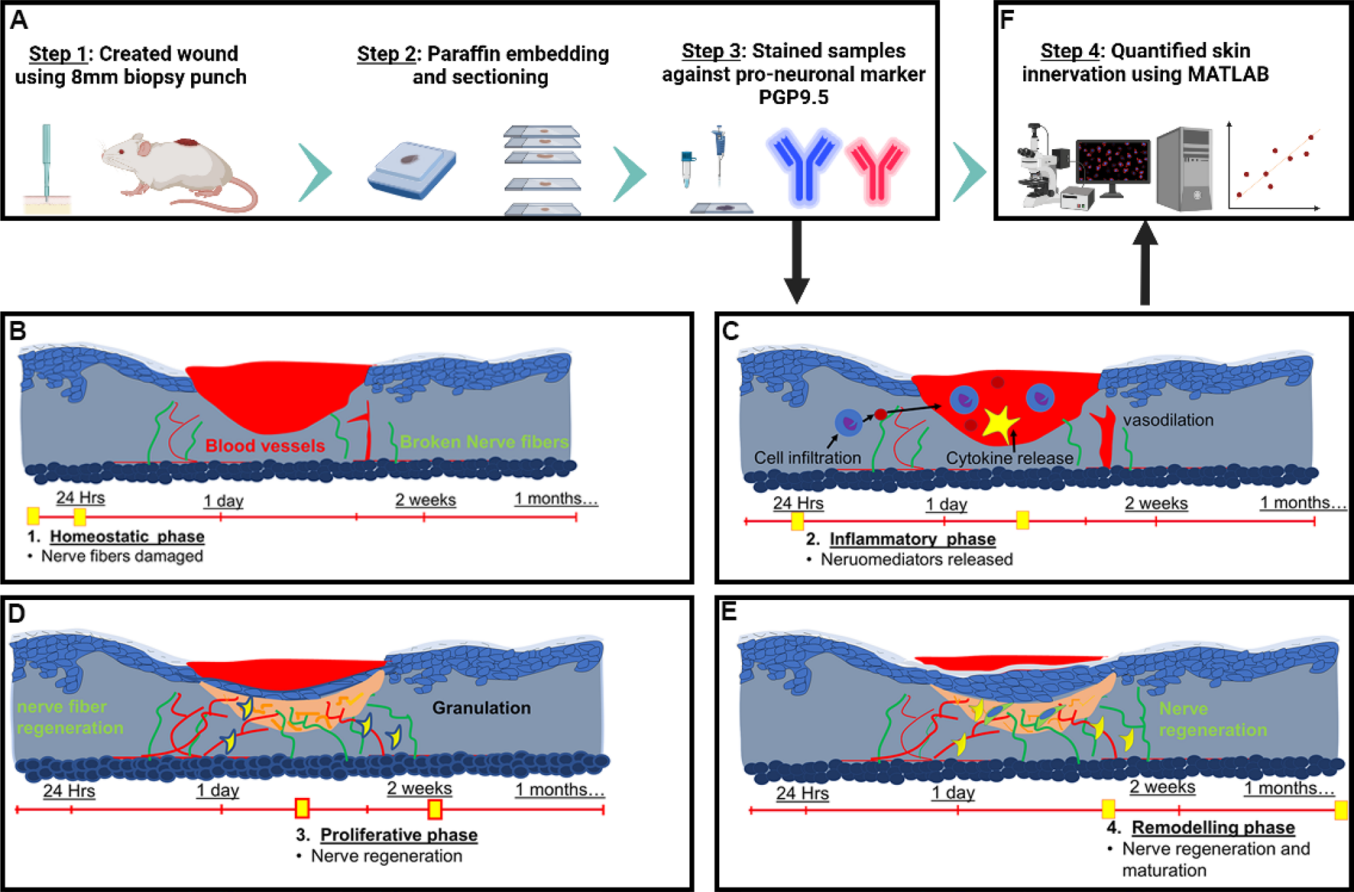
The experimental design and schematic depicting the methodology used to quantify skin innervation. (**A**) A biopsy punch of 8 mm in diameter is used to create the wound, and skin samples are collected and fixed on days 3, 7, 10 and 15. After fixation, the wounded tissue is paraffin-embedded and sectioned (5 μm thickness) for immunofluorescence analysis against PGP9.5 protein, a neuron-specific marker. (**B**–**E**) Illustration portraying different stages of wound healing. (**B**) The homeostatic phase lasts a few hours during which nerve fibers in the wound bed are damaged followed by the (**C**) inflammatory phase that can last between hours and days. (**D**) The proliferative phase lasts a few weeks during which re-innervation might be initiated and (**E**) during the remodeling phase wound matures and can last between weeks to years. In our study, we chose to quantify skin innervation at days 3, 7, 10 and 15 as an attempt to cover all phases of wound healing. (**F**) The immunohistochemistry (IHC) samples are analyzed using automated Matlab-assisted tools aided by DnCNN-based image denoising. The images were created with BioRender.com.

Utilizing an automated Matlab-assisted tool aided with DnCNN we quantified skin innervation during wound healing stages at days 3, 7, 10 and 15 (Fig. 1). The data show a positive correlation between the increase in nerve fiber density and re-epithelization.

## Materials and methods

### Animals

All methods were approved and performed in accordance with the relevant guidelines and regulations of Institutional Animal Care and Use Committee at UC Davis, were reviewed and approved by the institution's IACUC and performed at the UC Davis Teaching and Research Animal Care Services (TRACS) facility. Male C57BL/6 (older than 28 weeks, 30–35 g) mice were obtained from Jackson Laboratory. The animals were acclimated for one week after transfer from the vendor to the UC Davis vivarium. All male mice used in this experiment were kept in the containment unit of the animal facility, housed in cages with free access to food and water. Mice would be excluded from the experiment when they were at the anagen phase of hair cycle due to variation of healing rate, or when they lost > 20% weight during the experimental period. The animals were randomly assigned to the treatment groups based on their weight prior to the surgery. All experiments were performed in a biosafety cabinet and were done in triplicate (n = 3). The study is reported in accordance with ARRIVE guidelines.

### Wounding

The animals were sedated with 3–5% isoflurane two days prior to wounding surgery and the dorsal surface was shaved. To remove the remaining hair depilatory cream was applied and removed within 5–10 s of application. For surgery, all mice were anesthetized with isoflurane. Buprenorphine (0.05 mg/kg) was administered subcutaneously prior to the wounding procedure. Iodine and ethanol wipes were used to sterilely prep experimental mice. An 8 mm sterile skin biopsy punch instrument was used to create full-thickness wounds on the dorsal skin as described previously^7,31^. In this study we used splinted wound model to avoid wound contraction^32,33^. At the end of the experiment, mice were euthanized by cervical dislocation under 5% isoflurane, and skin samples were collected on days 3, 7, 10, and 15 post-wounding.

### Immunohistochemical analysis of wound tissues

After fixation, the wound tissue was paraffin-embedded and sectioned (5 μm thickness) for immunohistochemical analysis as described previously^34,35^. Primary antibodies against PGP9.5 (Invitrogen, Catalog # PA5-29012), and β-III tubulin (Invitrogen, Catalog # MA5-16308) were used. Donkey anti-Rabbit IgG (H + L) highly Cross-Adsorbed Secondary Antibody, Alexa Fluor™ 594 (Invitrogen, Catalog # A21207), and donkey anti-Mouse IgG (H + L) Highly Cross-Adsorbed Secondary Antibody, Alexa Fluor™ 488 (Invitrogen, Catalog # A21202), respectively were used. VECTASHIELD^®^ Antifade Mounting Media with DAPI (Vector Laboratories, Catalog # H-1200-10) was used to stain nuclei. Slides were imaged with an Olympus FV3000 Confocal Laser Scanning Microscope (Shinjuku City, Tokyo, Japan) as described previously^36–38^ and analyzed using Matlab 2021Image processing.

### Quantification

In order to accurately quantify wound healing, several steps are taken to ensure precision and accuracy in counting PGP9.5 positively stained pixels. One of the most important steps is preprocessing the images to remove any unwanted noise or artifacts that could potentially interfere with the analysis. Many factors can contribute to image noise, including non-specific staining, autofluorescence, equipment malfunctions, motion blur, and environmental factors. To eliminate noise from the images, a deep neural network known as the DnCNN network is employed. It is a convolutional neural network (CNN) specifically designed for image denoising^29^. It works by learning to map between noisy images and clean images and then use this mapping to remove noise from new images. The DnCNN network is trained on a large dataset of noisy and clean images and has shown effectiveness in a wide range of applications, including medical imaging^30^.

The DnCNN employed in this study is a trained model derived from the deep network training in MATLAB. Utilizing the Deep Learning Toolbox's pretrained DnCNN model, we integrated it within the MATLAB environment and invoked it through the MATLAB Deep Learning Toolbox. Our process involved denoising images by employing the DnCNN model from the Deep Learning Toolbox, ultimately producing denoised outputs. DnCNN effectively addresses this by enhancing capacity and flexibility for exploiting image attributes. Moreover, it can accelerate training and enhance denoising outcomes. Additionally, DnCNN, grounded in trained data, can discern pixels to filter and retain, thereby eliminating the need for manual adjustments across diverse images.

Once the images have been denoised, the next step is to count the PGP9.5 positive pixels in the images. Positive pixels representing innervation were identified by their immunoreactivity to PGP9.5 antibody (red color). However, identifying those pixels can be challenging because of the variation in the intensity of fluorescence due to different wound depths, healing stages, expression levels and sizes of expression areas. Additionally, the background intensity can pollute the pixel value counted as nerve fibers and nerve terminals. To overcome these challenges, we calculate the background value. This is achieved by identifying the low spectrum values of pixels and removing outliers. The background value serves as a reference point for subsequent analyses. By subtracting the background value from all pixels in the image, we effectively normalize the pixel values, making them more comparable across different images. This normalization step is essential for accurate comparisons and ensures that variations in background intensities do not influence our results. Next, we employ a robust method for identifying and omitting outliers from the image. We utilize a percentile-based approach where we focus on the 5 percent of data points that have the longest distance from the median. This 5 percent threshold has been carefully adjusted based on the specific characteristics of the image under analysis. It allows us to address the unique properties and variations in pixel intensity that may exist in different types of images. If a pixel value falls within this 5 percent range, it is considered an outlier and is excluded from further analysis. With the fluorescence threshold in place, we proceed to count the number of PGP9.5-positive pixels in the image. Each pixel's (red fluorescence value) is compared to the threshold, and any pixel with a value above the threshold is considered a positive neurite containing pixel count. This process is repeated for all the pixels in the image, determined together as the total number of neurites containing pixels. Next, to identify nerve fiber density in a target region of interest we divided the total number of positive pixels by the corresponding area. In addition to measuring nerve fiber density in the whole wound, we also analyzed the density of nerve fibers (positive pixels) separately for both the epidermis and dermis to get a spatial understanding.

### Reepithelization

After fixation, the wound tissue was paraffin-embedded, sectioned to 5 μm and H&E stained for determination of wound re-epithelialization as described previously^31,35^. Briefly, BioRevo BZ-9000 inverted microscope (Keyence, Osaka, Japan) was used to image all the histological sections. Measurements were done by an investigator blinded to experiment with the BZ-II viewer and analyzer (Keyence, Japan). The absence of underlying adipose tissue and hair follicles defines wound edges and wound healing is determined by the re-epithelialization of the epidermis layer^33,39^. The outgrowth of the newly formed epidermis was tracked manually from the wound edges and the percentage of the combined length of the re-epithelialization to the total length of the wounds was calculated.

### Statistics

Statistical analysis was performed using an unpaired, two-tailed student *t*-test as described previously^40,41^. Data are expressed as mean  ± SD. A *P*-value less than 0.05 was considered statistically significant.

## Results

### PGP9.5 as a specific neuronal marker

Anti-PGP9.5 antibody labels UCHL1/PGP 9.5 protein in the tissue sections. The protein is highly conserved and localized in neurons and is considered as a pan-neuronal marker that stains both sensory as well as autonomic nerves^42,43^. To deduct background signal, we carried out experiments with negative control for every sample omitting the primary antibody (Supplementary Fig. 1). The background signals from the negative control are determined and used for generating a cutoff window for quantification of the true PGP9.5+ signals during MATLAB-based quantification.

PGP9.5 staining is used widely and considered the gold standard for labeling skin innervation^27,28,42,44^, so throughout the study, we quantified PGP9.5 immunofluorescence staining for the detection of nerve fibers.

### Automated image processing and quantification of skin innervation

**Figure 2:**
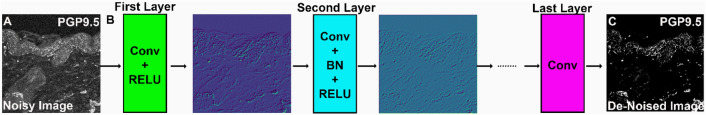
DnCNN network architecture for image denoising. (**A**) Noisy image as DnCNN input. (**B**) The DnCNN network architecture consists of multiple convolutional layers. Each convolutional layer includes batch normalization (BN), convolution (Conv), and rectified linear unit (ReLU) layers. The first layer takes the noisy image as an input, and the subsequent layers process the image to remove noise. (**C**) Output image after de-noising.

The quantification method used in this study employed an automated Matlab-assisted image analysis tool with a deep neural network to pre-process images and determine the range of skin innervation during different stages of wound healing. The use of the deep neural network ensured that noisy pixels did not affect the calculation of total neuronal coverage (Fig. 2). The depicted layers present how DnCNN operates, transforming a noisy wound image into a clearer and diagnostically valuable representation. Each layer embodies a distinct phase of noise reduction and feature enhancement, showcasing the model's capacity to discern between essential wound structures and undesirable noise. The first layer of the DnCNN model is responsible for capturing the low-level features of the image. During this stage, the initial noise present in the wound image is identified and basic filters are applied to smooth out the noise while preserving the main structures of the wound. As the image progresses through the second layer, which is part of the intermediate layers of the DnCNN model, more complex features and patterns are recognized, contributing to enhanced noise reduction and structure preservation. Each subsequent layer refines the denoising process, gradually enhancing the clarity of the image. The last layer of the DnCNN model is designed to further refine the denoised image and restore it closer to its original state. By this stage, most of the noise has been removed, and wound structures are significantly clearer. The final layer ensures that the denoised image maintains its clinical relevance by minimizing any artifacts introduced during the denoising process, thereby preserving the fidelity of the wound's clinical representation^29,45,46^.

Throughout the layers, the DnCNN model employs a combination of convolutional filters and nonlinear activation functions to transform the image and remove noise while preserving important features. The progressive nature of the layers enables the model to iteratively refine the denoising process, resulting in a cleaner and clearer wound image that can aid in accurate diagnosis and assessment^38,47,48^. Keep in mind that the specific appearance and effectiveness of the images after passing through each layer depend on the model architecture, training data, and noise characteristics. It's recommended to visually analyze the images to understand the improvements brought about by each layer.

Once the image has been processed by the DnCnn, our next step is to accurately identify the neurites using a statistical approach. If a threshold is too high, some neurites might not be detected, resulting in an underestimation of the neuronal coverage. Conversely, if the threshold is too low, non-neuronal elements in the image may be mistakenly identified as neurites, leading to an overestimation of neuronal coverage. To address this issue, we employed a statistical approach to determine the appropriate threshold value. First, we saved the R values of every pixel in the image to a list. We then calculated several statistical measures, including the minimum, maximum, mean, median, and interquartile range. Outliers were identified based on this statistical analysis and excluded from further consideration. To determine whether a pixel contained a neurite or not, we used the distance of the R-value from the third interquartile (Q3). Pixels with a closer distance to the maximum value in comparison to Q3 were selected as neurites. This method ensured that the threshold value was based on a statistically robust approach, which increased the accuracy of the quantification method.

Overall, the automated approach demonstrated in this paper required minimal manual intervention, making it suitable for large-scale studies and a reliable method for quantifying skin innervation during wound healing. Additionally, to demonstrate the significance of DnCNN and its impact on noise reduction, we compared two denoising techniques: the conventional threshold denoiser and the advanced DnCNN model (Supplementary Fig. 2). Using only conventional threshold denoiser, the nerve fiber density for uninjured skin (unwounded) at outer edge 1 of the wound is found to be 0.04 ± 0.003 pixels/mm^2 ^ with a density of 0.08 ± 0.03 pixels/mm^2^ found in the epidermis and 0.03 ± 0.007 pixels/mm^2^ in the dermis. Comparatively incorporating the DnCNN model the nerve fiber density for the same set of images is found to be 0.22 ± 0.03 pixels/mm^2^ with a density of  0.4 ± 0.1pixels/mm^2^ in the epidermis and 0.08 ± 0.02 pixels/mm^2^ in the dermis. The conventional threshold denoiser methods employ preset threshold values to detect and filter pixel noise and these approaches struggle to differentiate true noise pixels from low-value pixels, leading to potential misclassification. Further, setting threshold values for each image proves cumbersome. To minimize noise, we adjusted the threshold iteratively until background noise became imperceptible. As a result the threshold denoiser tends to inaccurately remove neurite positive pixels due to its indiscriminate approach resulting in underestimating the neurite pixel density. Conversely, the DnCNN required no such adjustment as the DnCNN, grounded in trained data, learns to discern pixels to filter and retain them (Supplementary Fig. 2). This highlights the advantage of the DnCNN in accurately maintaining crucial diagnostic information in wound images.

Furthermore, we tested our automated method to analyze neurite innervation for 30 µm-thick cross-sections. The nerve fiber density at dermis, epidermis and whole wound together showed non-significant change on comparing 5 µm-thick cross-sections to 30 µm-thick cross-section (Supplementary Fig. 3). Therefore, clearly demonstrating that the algorithm used in this study is efficient enough to detect neurites in 30 µm-thick cross-section of uninjured skin as well, where whole nerve fibers are easier to visualize but background can blur the quantification.

### Wounding reduced nerve fiber density

**Figure 3:**
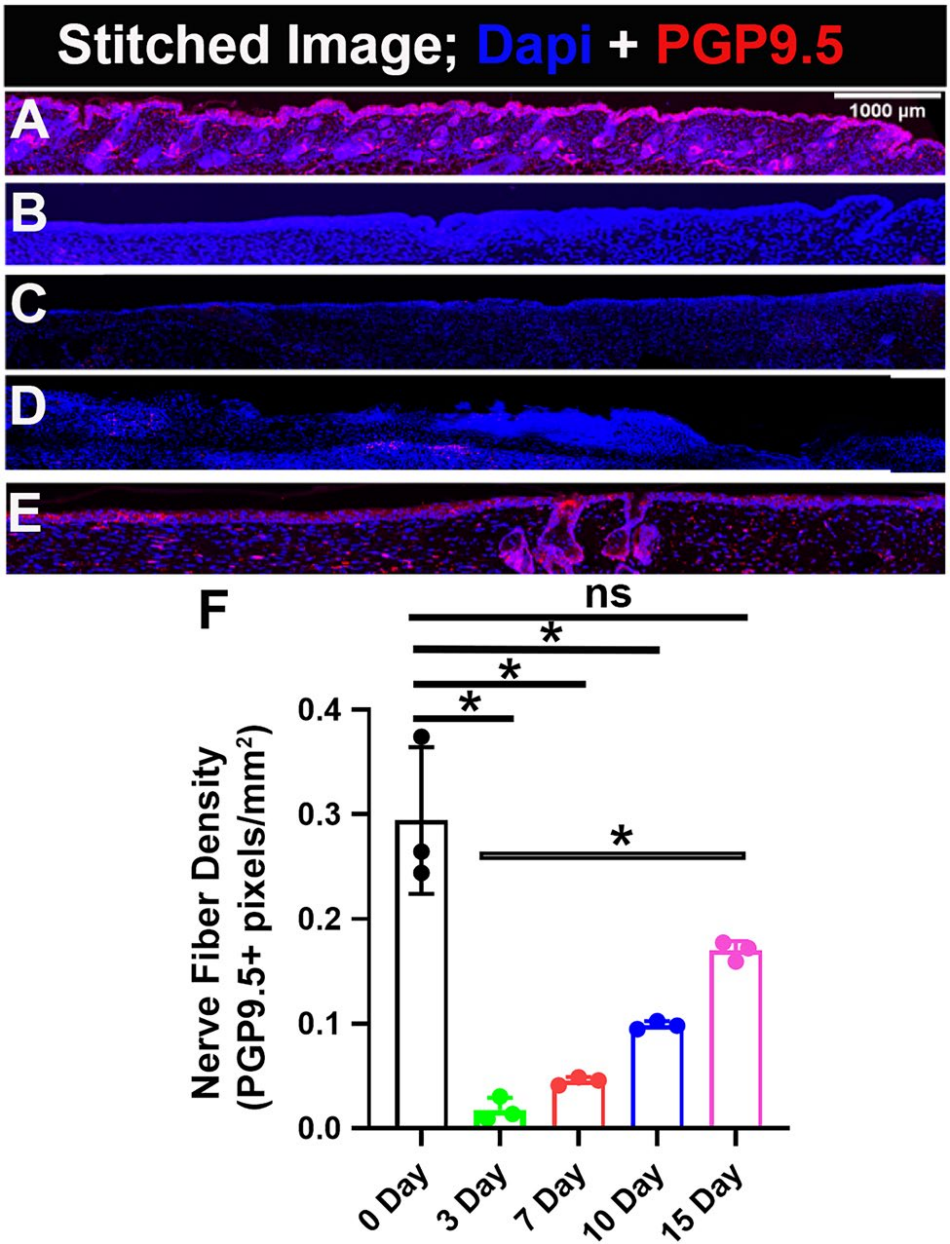
Gradual increase in Re-innervation in the wound bed. PGP9.5 is a pan-neuronal marker and DAPI stains nuclei. (**A**) Uninjured skin. Skin sample collected on (**B**) day 3, (**C**) day 7, (**D**) day 10 and (**E**) day 15 of wound healing. (**F**) Quantification of skin innervation for the whole wound bed represented as mean ± SD, n = 3 wounds from three mice in each group, *P < 0.05, ns is non-significant. The wound bed is recognized by the absence of hair follicles. Scale bar = 1000 μm.

**Figure 4:**
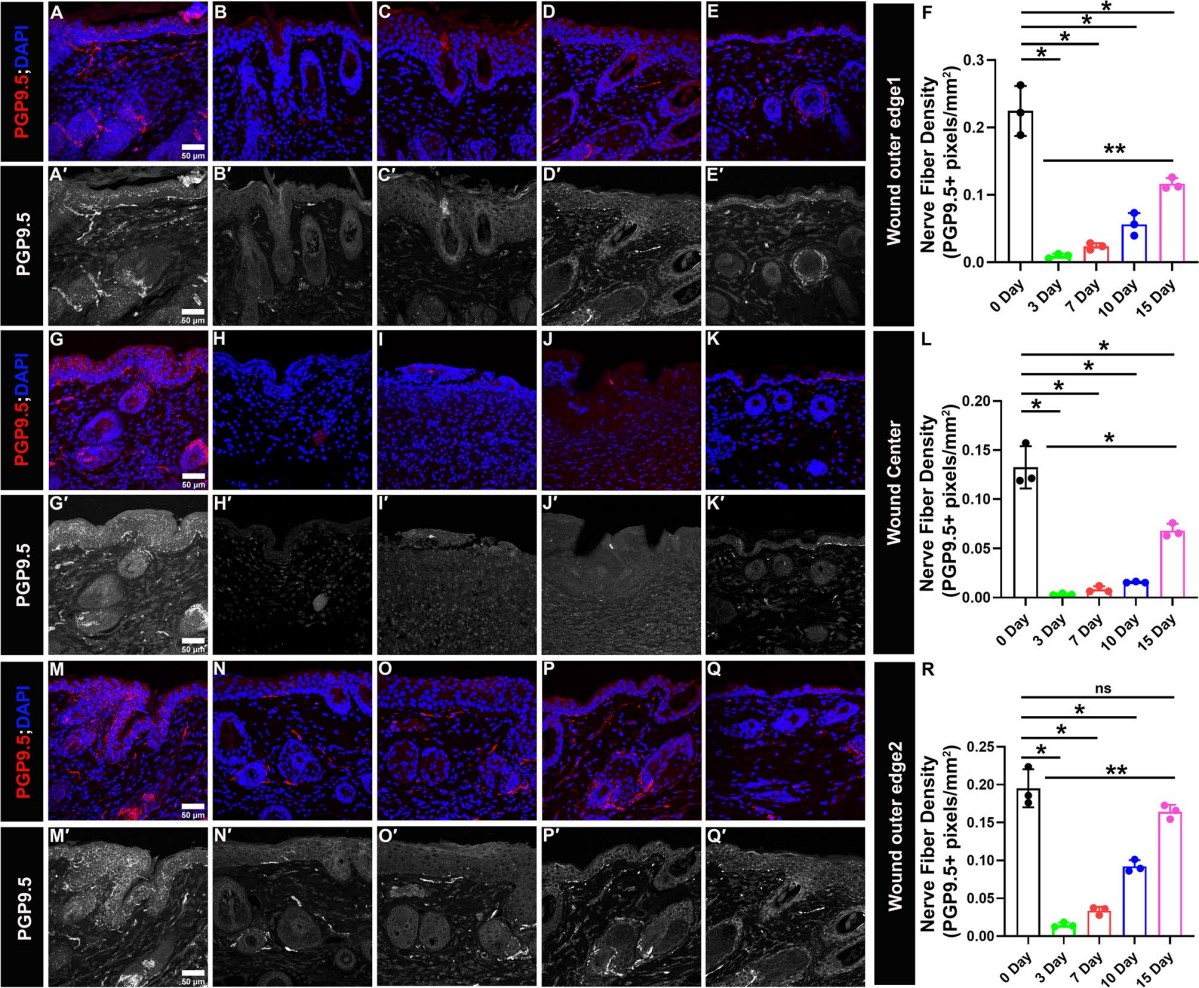
Gradual increase in Re-innervation at lateral wound edges and wound center. PGP9.5 is a pan-neuronal marker (in red) and DAPI stains nuclei of cell (in blue). (**A**′–**E**′, **G**′–**K**′, **M**′–**Q**′) Split images (in grey) show PGP9.5 immunoreactivity. (**A**–**E** and **A**′–**E**′) Immunoreactivity to PGP9.5 at wound outer edge 1 for skin samples. (**A**,**A**′) Uninjured, (**B**,**B**′) day 3, (**C**,**C**′) day 7, (**D**,**D**′) day 10, (**E**,**E**′) day 15. (**F**) Quantification of skin innervation at wound outer edge 1. (**G**–**K** and **G**′–**K**′) Immunoreactivity to PGP9.5 at wound center for skin samples. (**G**,**G**′) Uninjured, (**H**,**H**′) day 3, (**I**,**I**′) day 7, (**J**,**J**′) day 10, (**K**,**K**′) day 15. (**L**) Quantification of skin innervation at wound center. (**M**–**Q** and **M**′–**Q**′) Immunoreactivity to PGP9.5 at wound outer edge 2 for skin samples. (**M**,**M**′) Uninjured, (**N**,**N**′) day 3, (**O**,**O**′) day 7, (**P**,**P**′) day 10, (**Q**,**Q**′) day 15. (**R**) Quantification of skin innervation at wound outer edge 2. All quantification data are represented as mean ± SD, n = 3 wounds from three mice in each group, *P < 0.05, **P < 0.001, ns is non-significant. Scale bar = 50 μm.

Immunoreactivity was detected in intraepidermal and dermal nerve fibers and cells. The positively stained nerve fibers are quantified for the whole wound bed (Fig. 3; Table 1) and separately for the lateral wound boundaries, and the wound center (Fig. 4; Table 1). The nerve fiber density individually for the epidermis and dermis is also quantified respectively (Supplementary Fig. 4; Table 1). The nerve fiber density for uninjured skin (unwounded) is found to be 0.29 ± 0.07 pixels/mm^2^ for which a density of 0.64 ± 0.21 pixels/mm^2^ is found in the epidermis and 0.27 ± 0.07 pixels/mm^2^ in the dermis. Compared to uninjured skin, as expected after creating a wound, there is a considerable reduction in nerve fiber density throughout the wound bed (WB) (Fig. 3; Table 1).

**Table 1:** Nerve fiber density (pixels/mm^2^) at wound bed, wound outer edge 1, wound center, and wound outer edge 2 on day 0, 3, 7, 10 and 15 of healing.

Epidermis					Dermis					Whole wound				
0 Day	3 Day	7 Day	10 Day	15 Day	0 Day	3 Day	7 Day	10 Day	15 Day	0 Day	3 Day	7 Day	10 Day	15 Day
Wound bed														
0.64 ± 0.21	0.0 ± 0.0	0.0 ± 0.0	0.08 ± 0.004	0.44 ± 0.04	0.27 ± 0.07	0.02 ± 0.01	0.04 ± 0.003	0.10 ± 0.004	0.16 ± 0.007	0.29 ± 0.07	0.02 ± 0.01	0.045 ± 0.004	0.098 ± 0.004	0.169 ± 0.009
Wound outer edge 1														
0.4 ± 0.1	0.01 ± 0.01	0.04 ± 0.01	0.1 ± 0.04	0.2 ± 0.02	0.08 ± 0.02	0.004 ± 0.001	0.01 ± 0.001	0.01 ± 0.002	0.05 ± 0.003	0.22 ± 0.04	0.01 ± 0.003	0.023 ± 0.005	0.06 ± 0.024	0.12 ± 0.008
Wound center														
0.2 ± 0.02	0 ± 0	0 ± 0	0 ± 0	0.09 ± 0.02	0.13 ± 0.02	0.003 ± 0.001	0.009 ± 0.003	0.016 ± 0.001	0.066 ± 0.01	0.132 ± 0.021	0.003 ± 0.001	0.008 ± 0.003	0.015 ± 0.001	0.07 ± 0.01
Wound outer edge 2														
0.28 ± 0.07	0.03 ± 0.004	0.06 ± 0.006	0.15 ± 0.011	0.243 ± 0.018	0.13 ± 0.01	0.006 ± 0.002	0.009 ± 0.004	0.04 ± 0.01	0.10 ± 0.003	0.19 ± 0.025	0.01 ± 0.003	0.03 ± 0.005	0.092 ± 0.0085	0.16 ± 0.009

### Innervation gradually appears and reaches a level close to intact skin—15 days after wounding

On the day 3 of wound healing, nerve fiber density in the wound bed is found to be 0.02 ± 0.01 pixels/mm^2^ which is significantly less than the unwounded skin (0.29 ± 0.07 pixels/mm^2^). The nerve fiber density reported (0.02 ± 0.01 pixels/mm^2^) is found only in the dermis, yet the epidermis has not regenerated in the wound bed, so the intraepidermal nerve fiber density value is 0. On day 7, the nerve fiber density increases slightly to 0.045 ± 0.004 pixels/mm^2^. The intraepidermal nerve fiber density is still 0. The wound sections on day 10 show some traces of intraepidermal nerve fibers, the nerve fiber density quantified is 0.080 ± 0.005 pixels/mm^2^. The nerve fiber density in the dermis, 0.10 ± 0.004 pixels/mm^2^, also steadily shows an increasing trend. The nerve fiber density for the whole wound bed is 0.098 ± 0.004 pixels/mm^2^ on day 10, which is still significantly less compared to unwounded skin (0.29 ± 0.07 pixels/mm^2^). Interestingly, on day 15 of healing there is a huge increase in intraepidermal nerve fiber density, 0.44 ± 0.04 pixels/mm^2^. The nerve fiber density in the dermis also substantially increases to 0.16 ± 0.007 pixels/mm^2^. On day 15, the changes in nerve fiber density for the whole wound bed compared to unwounded skin (0.17  ± 0.009 pixels/mm^2^ vs 0.29  ± 0.07 pixels/mm^2^) are non-significant (Fig. 3 and Supplementary Fig. 4) and interestingly significant when compared to day 3 of healing ( 0.17  ± 0.009 pixels/mm^2^ vs 0.02 ± 0.01 pixels/mm^2^). The data narrates that there is a gradual increase in nerve fiber density through the time series of wound healing, and there is a significant re-innervation and values start reaching close to normal from day 15 of healing onwards.

### The wound center and wound edge show a similar trend in re-innervation

We also quantified nerve fibers separately for wound edges and wound center on days 3, 7, 10, and 15 of wound healing and compared it with the innervation of unwounded skin (Fig. 4 and Supplementary Fig. 4; Table 1). For unwounded skin the density values obtained at outer edge 1 are: intraepidermal (IE) = 0.4 ± 0.1 pixels/mm^2^; dermis (D) = 0.08 ± 0.02 pixels/mm^2^; whole unwounded skin = 0.22 ± 0.03 pixels/mm^2^. It is evident that on day 3, 7 and 10 there is a significant decrease in the IE and D nerve fiber density compared to uninjured skin (Table 1). As expected, the nerve fiber density steeply increased on day 15 of healing: IE = 0.2 ± 0.02 pixels/mm^2^; D = 0.05 ± 0.003 pixels/mm^2^; whole wound outer edge 1 = 0.12 ± 0.008 pixels/mm^2^. The values for IE and D show non-significant change compared to uninjured skin except for whole wound outer edge 1 where nerve fiber density is still significantly less compared to uninjured skin (0.12 ± 0.008 pixels/mm^2^ vs 0.22 ± 0.03 pixels/mm^2^). Also, the change found is significant compared to 3 days for IE, D and whole wound outer edge 1  (Fig. 4 and Supplementary Fig. 4; Table 1). Therefore, data signify that at outer edge 1 of the wound, a considerable level of re-innervation takes place up to day 15 of healing. However, total nerve fiber density cannot reach the normal level i.e. the values are significantly less compared to the uninjured skin. This is contrary to outer edge 2, where significant reinnervation happens and total nerve fiber density at day 15 also show non-significant change compared to uninjured skin at IE, D, and whole wound outer edge 2 (Fig. 4 and Supplementary Fig. 4; Table 1). Whereas, the wound center behaves like outer edge 1 where a significant re-innervation happens by day 15 but the nerve fiber density compared to uninjured skin (0.07 ± 0.007 pixels/mm^2^ vs 0.13  ± 0.02 pixels/mm^2^) show significantly lower values. Stating that a considerable increase in nerve fibers at the wound outer edge 1 and wound center is still to be expected after day 15.

### Re-innervation of the wound correlates strongly with re-epithelialization

**Figure 5:**
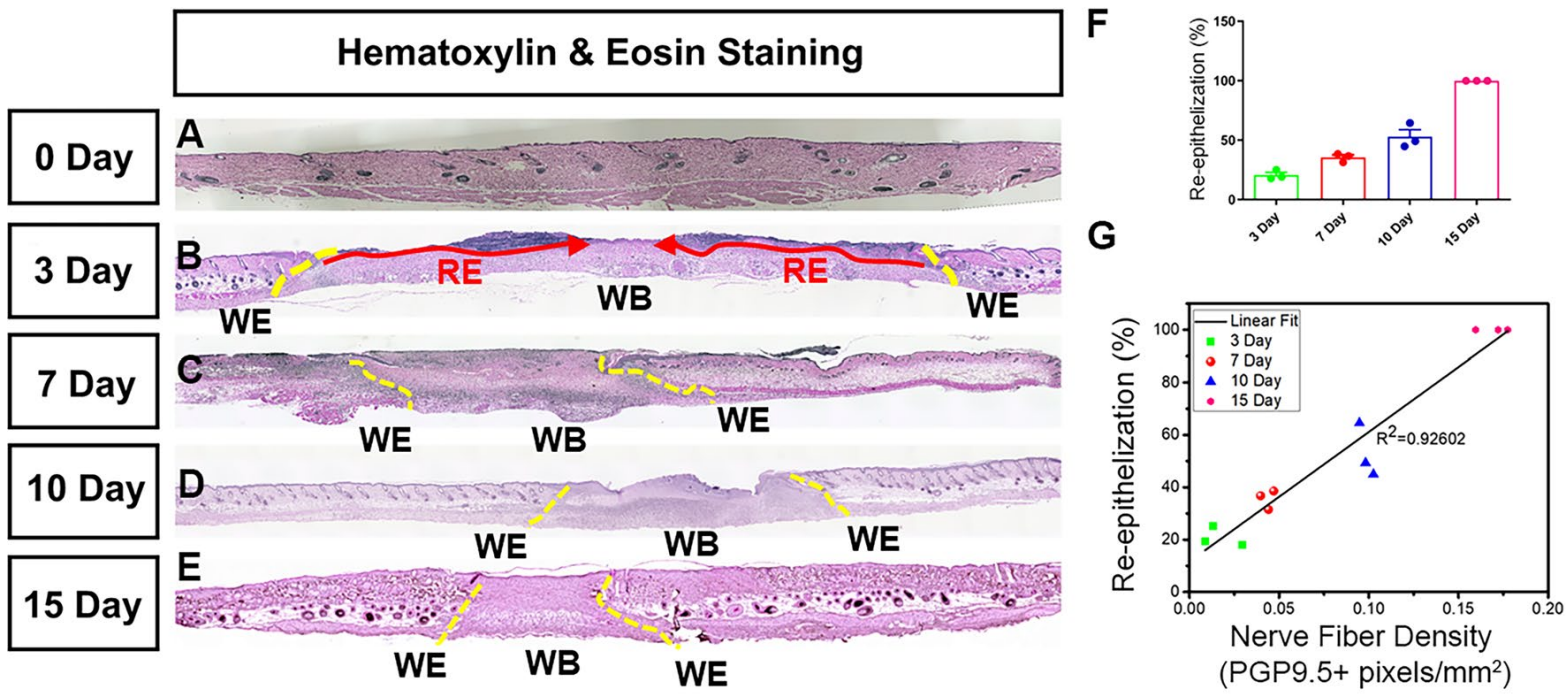
Positive correlation between re-innervation and re-epithelization. A representative image of H&E staining of the skin sample collected on (**A**) day 0, (**B**) day 3, (**C**) day 7, (**D**) day 10, (**E**) day 15. The original wound edge (yellow dashed lines) on each side is determined by the absence of subdermal adipose tissue. Re-epithelialization (red arrows) is defined by epithelial cell growth. (**F**) quantification of re-epithelization, (**G**) correlation between nerve fiber density and re-epithelization at day 3, 7, 10 and 15 of wound healing. R^2^ = 0.926 show strong positive relation. *WE* wound edge, *WB* wound bed, *RE* re-epithelization.

Denervation has a detrimental effect on cutaneous wound healing. Severing the nerves hinders cutaneous wound healing, and sympathetic denervation of the skin delays re-epithelization^10^. Re-epithelization is defined by the epithelial cells migrating and growing over the wound bed and complete epithelial covering of the wound is a criterion to evaluate if a wound has healed properly^31,49^. We investigated the correlation between nerve fiber density and re-epithelization on days 3, 7, 10, and 15 of healing and found a strong correlation (R^2^ = 0.926) between the two (Fig. 5). This corroborates our hypothesis that the regeneration of nerve fibers is critical for proper wound healing in time.

## Discussion

The skin is densely innervated with a complex architecture and network of cutaneous nerves, which are present in both epidermis and dermis. The degree of innervation has a direct effect on all the overlapping stages of wound healing^1^, and previously it has been reported that denervated wounds take a longer time to heal^50^. Therefore, precise quantification of nerve fiber density during different stages of wound healing becomes critical. PGP9.5 staining is considered as a gold standard for the quantification of nerves in skin samples in mammals^42,44^. However, precise quantification of cutaneous nerves is challenging because of background noise and a different pixel intensity of PGP9.5+ neurons throughout epidermis and dermis. Therefore, to solve the problem we used the deep neural network DnCNN, for pre-processing (de-noising) of the IHC-images, followed by stringent statistical methods to formulate a threshold boundary, which is broad enough to include all the features of interest and strict enough to exclude background, so that PGP9.5+ pixels are precisely quantified in cutaneous wounds.

Applying our newly developed technique, automated Matlab-assisted tool aided with the DnCNN, we quantified skin innervation in Female C57BL/6 mice during normal wound healing at days 3, 7, 10 and 15. The data show that (1) the skin wound causes substantial reduction in nerve fiber density, which significantly increased by day 15 of wound healing, (2) one of the wound edge and wound center still have significantly less nerve fiber density compared to uninjured skin at day 15 of wound healing, (3) re-epithelization and innervation share a strong correlation (R^2^ = 0.926).

We found that on day 3 and day 7 nerve fiber density is almost negligible when compared to the unwounded skin (Fig. 3; Table 1). From day 10 onwards the nerve fiber density starts increasing marginally but reaches a significant level at the later stage of wound healing on day 15. At day 15, comparing the nerve fiber density for the whole wound bed or individually for the epidermis as well as dermis shows non-significant change when compared to the uninjured skin for the respective skin regions. Moreover, interestingly the values are significantly higher when compared to the initial stages of wound healing, day 3, at respective skin regions (Fig. 3; Supplementary Fig. 4; Table 1). This trend together suggests that re-innervation of the wound is initiated at the later stages of wound healing and starts becoming substantial from day 15 onwards during normal wound healing conditions in mammals. The data seems logical because for re-innervation, proliferation of neuronal cells (Schwann cells) needs to be initiated, followed by orchestrated phenomena to develop new cutaneous nerves^51–55^, and the proliferation stage of neuronal cells (Schwann cells) might overlap with the proliferation stage of wound healing, which begins approximately at day 3 and lasts for a couple of weeks. Thus, re-innervation also starts appearing substantially from day 10 onwards and shows a significant increase by day 15. Quantifying nerve fiber density separately for wound edges and wound center showed an interesting trend. At outer edge 2, similar to the whole wound, the nerve fiber density at day 15 showed a non-significant change compared to uninjured skin at IE, D, and for the whole wound outer edge 2 (Fig. 4 and Supplementary Fig. 4; Table 1). However, for the outer edge 1 and wound center nerve fiber density cannot reach the pre-injury level i.e., the values are still significantly less compared to uninjured skin for the whole wound (Fig. 4). This suggest that nerve fibers are continuously innervating the cutaneous wound even beyond day 15 of wound healing. Also, on day 15 of healing the nerve fiber density at outer edge 1 is less compared to outer edge 2 (0.12  ± 0.008 pixels/mm^2^ vs 0.16 ± 0.009 pixels/mm^2^) (Fig. 4). This could be due to the difference in distance between the two outer edges of the skin wound from the cell bodies located in the dorsal root ganglia from where the cutaneous sensory nerves originate^56^. Additionally, we have been able to find a strong correlation (R^2^ = 0.926) between re-epithelization and nerve fiber density during time-series of wound healing, which not only corroborates the fact that the regeneration of nerve fibers is critical for proper wound healing in time but also validated our technique of using automated Matlab-assisted tool aided with DnCNN for denoising to precisely capture PGP9.5+ pixels, and thus calculate nerve fiber density.

## Conclusion and future directions

This study demonstrates the use of automated deep-learning tools to accurately quantify skin innervation within the wound area, including the wound bed, wound center, and wound edges in mammals. By effectively reducing noise in immunohistochemistry (IHC) images, this technique allows for precise quantification of nerve fiber density in the epidermal and dermal layers of the wound region (bed, center, and edges). This data-centric approach provides crucial insights that can contribute to creating predictive models for applications in precision medicine in wound healing.

The statistical approach introduced in this study serves as a valuable alternative to previously described methods^20–26^. It eliminates the need for manual data labeling, a requirement in traditional machine learning approaches for generating training data. The denoising method employed in this study has already undergone training using biological images, obviating the necessity for further training on wound-specific images. As a result, researchers can save considerable time and resources that would otherwise be expended on data collection and annotation.

The data generated shows that there is a gradual increase in nerve fiber density throughout the wound bed as well as at the wound edges, with maximum value reaching on day 15 that indicates a significant trend in re-innervation when compared to day 3 of wound healing. Additionally, on day 15 of healing nerve fiber density for the whole wound bed, and at wound edge 2 reaches close to the uninjured skin. However, at wound outer edge 1 and the wound center the total nerve fiber density at day 15 of healing is still significantly less compared to the uninjured skin asserting that re-innervation is still a continuous process beyond day 15 of healing and is important for the complete healing of the wound. The correlation between the increase in nerve fiber density and re-epithelization further supports the importance of cutaneous nerve fibers in wound healing. Overall, while our method does not elucidate the morphological characteristics of cutaneous nerves in three dimensions, it does offer a simple and cost-effective way to analyze the variations in skin innervation during different stages of wound healing.

Numerous treatments aimed at expediting and enhancing wound healing have been documented. Electric stimulation, for example, has shown promising results in promoting innervation in human cutaneous wounds^57–59^. Many bandages e.g., Procellera claim to deliver electric fields to promote wound healing^59^. Our approach and methodology developed in this paper can assist the quantitative determination of nerve fiber density in space through the time of wound healing and facilitate assessing the effects on wound treatment. This high throughput method can be adopted for the quantification of innervation in various skin pathologies in addition to wound healing, and for the quantification of innervation of other tissues and organs.

### Supplementary Information

Supplementary Figures.

## Data Availability

The authors confirm that the data supporting the findings of this study are available within the article or its supplementary materials.
