# Supplementary material for: Quantifying innervation facilitated by deep learning in wound healing
Source: Sci Rep. 2023 Oct 6;13:16885. doi: 10.1038/s41598-023-42743-5 (PMC10558471; doi:10.1038/s41598-023-42743-5)
Supplement: Supplementary file 1 — Supplementary Figures. [file 41598_2023_42743_MOESM1_ESM.docx]

**Quantifying innervation facilitated by deep learning in wound healing**

Abijeet Singh Mehta^1,2,^*, Sam Teymoori^3^, Cynthia Recendez^1,2^, Daniel Fregoso^1^, Anthony Gallegos^1^, Hsin-Ya Yang^1^, Elham Aslankoohi^4^, Marco Rolandi^4^, Roslyn Rivkah Isseroff^1^, Min Zhao^1,2,^*, Marcella Gomez^3,^*

*Co-corresponding authors

ASM: [abijeet.mehta@northwestern.edu](mailto:abijeet.mehta@northwestern.edu)

MZ: [minzhao@ucdavis.edu](mailto:minzhao@ucdavis.edu)

MG: [mgomez26@ucsc.edu](mailto:mgomez26@ucsc.edu)

^1^ Department of Dermatology, University of California, Davis, CA, USA, 95616

^2^ Department of Ophthalmology, University of California, Davis, CA, USA, 95616

^3^ Department of Applied Mathematics, University of California, Santa Cruz, CA, USA, 95064

^4^Department of Electrical and Computer Engineering, University of California, Santa Cruz, CA, USA, 95064

**Supplementary Figures**

**Supplementary Figure 1**


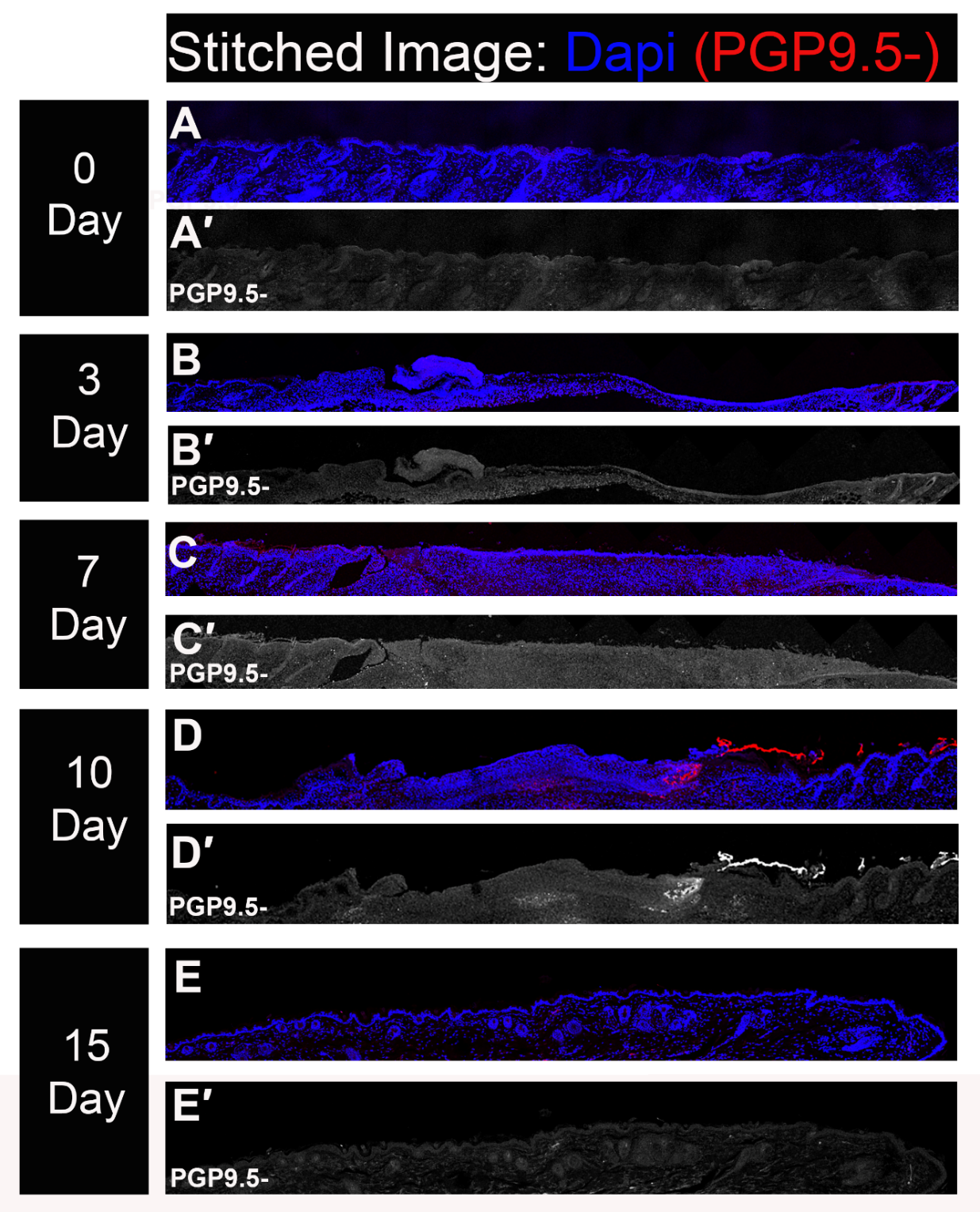


**Supplementary Figure 1: Stitched Immunohistochemistry images of 5 μm vertical sections of punch biopsies as a negative control for PGP9.5.** PGP9.5 is a pan-neuronal marker and DAPI stains the nuclei (In blue). (A) Uninjured skin. Skin samples were collected on (B) day 3 (C) day 7 (D) day 10 (E) day 15. The red color is autofluorescence from a dead skin flap.

**Supplementary Figure 2**

**
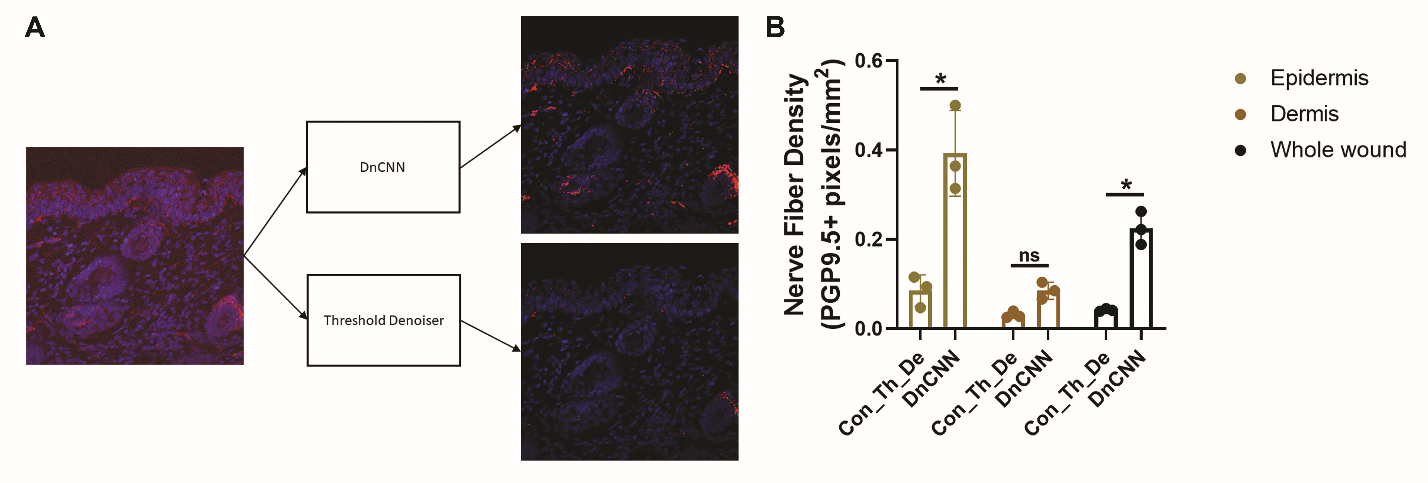
**

**Supplementary Figure 2: Comparison between conventional threshold denoiser and the advanced DnCNN model.** (A) PGP9.5 staining (in red) at outer_edge 1 of uninjured skin and subsequent denoising of stained image using DnCNN model compared to threshold denoiser (B) Quantification of innervation in epidermis, dermis and whole wound of uninjured skin outer_edge 1 using conventional threshold denoiser (Con_Th_De) and advanced DnCNN model. All quantification data are represented as mean ± SD, n = 3 wounds from three mice in each group, *P < .05, ns= non-significant.

.

**Supplementary Figure 3**

**

**

**Supplementary Figure 3: Comparison between 5μm thick and 30μm thick section.** (A) PGP9.5 staining at outer_edge 1 of uninjured skin for 30μm thick section. (B) Quantification of innervation for 5μm and 30μm thick section in epidermis, dermis and whole wound at outer_edge 1 of uninjured skin. All quantification data are represented as mean ± SD, n = 3 wounds from three mice in each group, ns= non-significant.

**Supplementary Figure 4**


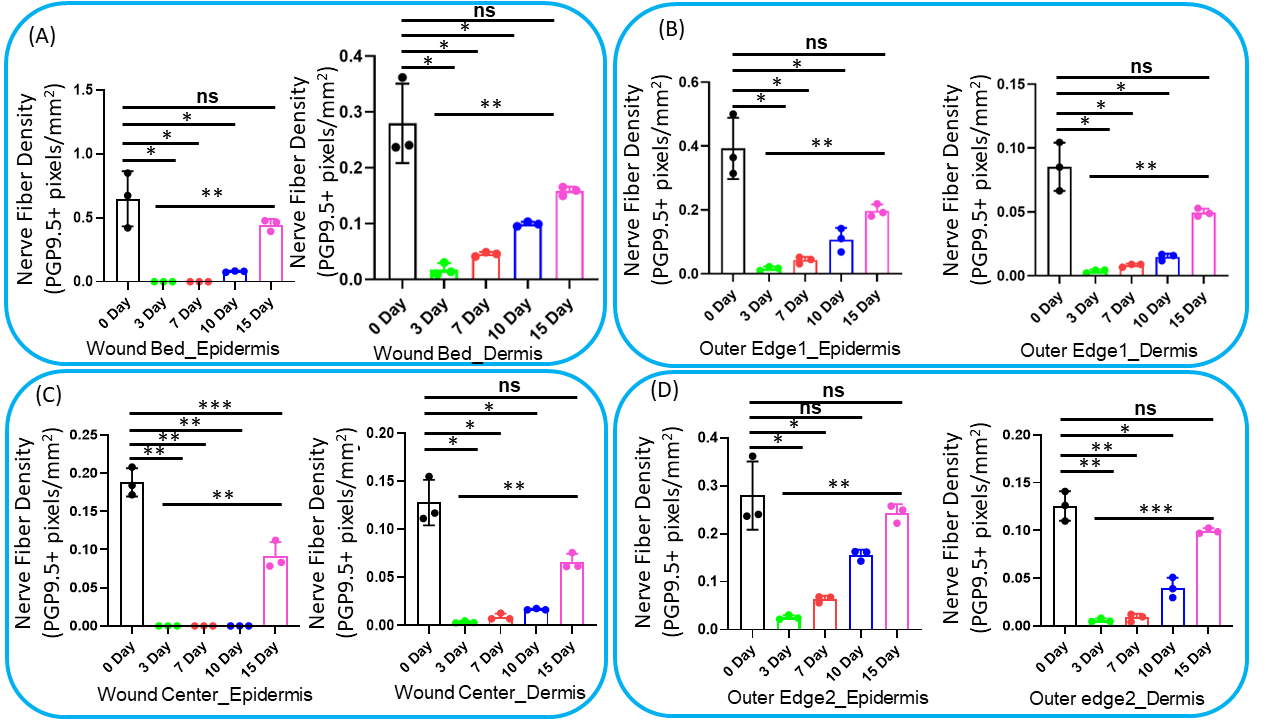


**Supplementary Figure 4: Intraepidermal and dermis innervation.** Quantification of innervation in epidermis and dermis for (A) Wound bed (B) Wound outer edge 1 (C) Wound center (D) Wound outer edge 2. All quantification data are represented as mean ± SD, n = 3 wounds from three mice in each group, *P < .05, **P < .001, ***P < .0001, ns is non-significant.
